# Long-term results and quality of life after vibrant soundbridge implantation (VSBs) in children and adults with aural atresia

**DOI:** 10.1007/s00405-023-08100-y

**Published:** 2023-08-21

**Authors:** Sophia Gantner, Alexandra Epp, Marlene Pollotzek, John Martin Hempel

**Affiliations:** 1 Department of Otorhinolaryngology, Head and Neck Surgery, Ludwig Maximilian University Munich, Munich, Germany; 2 Paediatric Hospital, University Hospital, Augsburg, Germany

**Keywords:** Vibrant soundbridge (VSB), Active middle ear implants (AMEI), Aural atresia, Conductive hearing loss, Rehabilitation

## Abstract

**Purpose:**

The aim of this study was to evaluate the long-term effectiveness and acceptance of the active middle ear implant system Vibrant Soundbridge (VSB^®^, MED-EL, Austria) in patients with aural atresia or aplasia (children and adults).

**Methods:**

Data from 51 patients (mean age 13.9 ± 11.3 years), 42 (79.2%) children and adolescents, and 11 (20.8%) adults) who received a VSB implant between 2009 and 2019 at the Department of Otolaryngology at LMU Clinic Großhadern, Munich were included in the study. Pure-tone audiometry, speech recognition in a quiet environment and in a noisy environment were performed preoperatively, during the first fitting of the audio processor, after 1–3 years, after 3–5 years, and after 5 years (if possible). The follow-up period ranged from 11 to 157 months with a mean of 58.6 months (4.8 years). Furthermore, the benefit of the VSB was evaluated by self-assessment questionnaires (Speech, Spatial, and Qualities of Hearing Scale, respectively, for parents).

**Results:**

Significant improvements were observed in hearing and speech comprehension immediately after the initial fitting of the VSB system (mean hearing gain 38.4 ± 9.4 dB HL) and at follow-up intervals (1–3, 3–5 and after 5 years) for children and adults (*p < *0.01). The values remained stable over the long-term, indicating a sustained functional gain from the VSB (mean hearing gain 38.9 ± 9.2 dB HL). The results of the self-assessments affirm the positive influence on hearing and speech comprehension with the VSB. With the VSB, there was an improvement of 41.3 ± 13.7% in the Freiburg monosyllable test.

**Conclusion:**

These results (a stable hearing gain over the long term, a good tolerance of the implant and an improvement in quality of life) affirm the recommendation for using the active middle ear implant VSB as early as permitted for aural atresia and aplasia patients. This study represents the audiometric results with the (to date) largest collective of aural atresia patients and with a long follow-up period.

**Supplementary Information:**

The online version contains supplementary material available at 10.1007/s00405-023-08100-y.

## Introduction

The absence of an open external auditory ear canal, called aural atresia (AA), can be congenital and present as hypoplasia or aplasia of the external auditory canal. It is often associated with dysmorphic formation of the auricle, the middle ear bones, and the inner ear structures, but it can also be acquired in periods in later life, for example, caused by an inflammation or trauma [1]. Aural atresia may be unilateral or bilateral, and results in (besides possible cosmetic impairment) conductive hearing loss or, in some cases with the addition of an inner ear impairment, mixed hearing loss. Independent of the onset of unilateral hearing loss (congenital or acquired), associated difficulties in directional hearing or speech recognition in noisy environments can affect the quality of life [2]. In particular, children with congenital aural atresia may have impairments in speech and language development, and consequently may perform poorly in school [3]. There are studies that show a positive relationship between the time of first hearing amplification by hearing devices, compliance in aid use, speech and language abilities, and general development of children [3]. Children with AA who receive early amplification have similar language, communication, reading, and school performance to normal-hearing classmates [4]. Consequently, early treatment of hearing impairment is recommended in various national and international guidelines [1, 5–7]

Possibilities for treatment and rehabilitation are individually determined and include surgical correction of the outer and middle ear, bone conduction devices, or active middle ear implants (AMEI) [8]. These implants have shown a benefit for patients with sensorineural hearing loss for which conventional hearing aids are not sufficient, and can also be a good option for patients with atresia or microtia [9].

The Vibrant Soundbridge (VSB, Vibrant MED-EL, Innsbruck, Austria) is a semi-implantable active middle ear implant system. It consists of a floating mass transducer (FMT), which receives the signals and transduces them into mechanical vibrations, and an external audioprocessor, which contains the microphone and is responsible for signal processing. The programming settings of the audioprocessor are adjusted in accordance with the hearing loss of the person. The signal is transmitted through the skin to the VORP. The electromagnetic FMT of the implant reproduces and augments the vibratory motion of the ossicular chain [10]. Currently, children must be aged 5 or above to be eligible for this treatment.

The FMT can be attached to the long or the short process of the incus [11], on the round window membrane [12, 13], on the head of the stapes, or on the oval window [14], or it may be combined with a passive middle ear prosthesis [15], depending on the anatomical and pathological conditions of the middle ear. All the coupling points provide satisfactory hearing improvements [11, 16]. The functional gain with the VSB typically ranges between 30 and 35 dB [12, 13, 17].

Compared to the indications mentioned above, for which there are many long-term studies on satisfaction and hearing gain, there are not many studies that describe the results in patients with aural atresia over long periods of time. The aim of the present study was to assess the long-term performance and satisfaction of children, adolescents and adults with congenital or acquired aural atresia, who have been wearing the VSB for up to 12 years (1 month–12 years postsurgery).

## Materials and methods

### Subjects

With approval from the local Ethics Committee (Ethikkommission der Medizinischen Fakultät der LMU München, Ref. 20–078), data from 51 patients (23 females, 26 males, age 13.8 ± 11.6 years) with unilateral or bilateral permanent conductive hearing loss due to aural atresia/aplasia who received a VSB implant at the Department of Otolaryngology at LMU Klinikum Großhadern between 2008 and 2019 were used for the study. Patients with hearing loss caused by chronic infections of the middle ear were excluded. As two patients had bilateral atresia of the auditory canal, 53 VSB implantations were performed in 51 patients.

### Study design

A retrospective and prospective longitudinal design was used in this study. Medical records including operation and laboratory reports and, in particular, the audiological data preoperatively, during the initial fitting, and during subsequent follow-up visits were analyzed. Since the follow-up examinations were not attended by the patients at the same intervals, the follow-up period was divided into time periods: initial fitting, 6–8 weeks after surgery, after 1–3 years, after 3–5 years, and after 5 years. After potential participants were identified (*n = *51), informed written consent for the study was obtained from all the participants or from their parents. In addition, self-assessment questionnaires were either handed out to the patients personally or sent by mail. All the methods were carried out in accordance with relevant guidelines and regulations.

### Audiological testing

The audiological data consisting of the results of frequency-dependent hearing threshold and audiological speech comprehension were evaluated before and after implantation as well as during regular follow-up examinations. Subsequently, the unaided and VSB-aided hearing thresholds were compared to calculate functional gain.

The following measurement methods were used in the examinations underlying this study:

- Pure-tone audiometry was used to determine the frequency-dependent hearing threshold and functional gain curve. Pure-tone audiometry was performed for each ear in the frequency range from 125 to 8000 Hz with a headphone for AC (audiometer model AT900, company Auritec, headphones DT48, company beyerdynamic). Bone conduction (BC) was recorded via a calibrated BC vibrator (bone conduction Radioear B71) held on the mastoid process. To avoid overhearing by the opposite ear, the better hearing ear was masked by narrowband noise. The functional gain curve was detected in free-field warble tone measurement at frequencies of 250 Hz–8000 Hz (in the unaided and VSB-aided condition). During the measurements, the contralateral ear was also masked by narrowband noise.
- Sound field audiometry was used to determine word recognition using the Freiburg monosyllabic speech test in a quiet environment at 65 dB SPL [18]. The Oldenburg sentence test (OLSA 10–68 years) and the Oldenburg sentence test for children (OLKISA 5–9 years), (an adaptive speech in noise test), were used to detect changes in the speech reception threshold [19]. In the first measurement, speech was presented from the front, and the background noise was presented from the side with normal hearing (S0°/N90° or 270°). In the second measurement, speech was presented to the hearing-impaired or VSB-supplied ear, and the noise to the normal-hearing/unprovided side (S270° or 90°/N45° or N315°). In patients with bilateral VSBs, both the speech and the noise came from the front (S0°/N0°).

Using these methods, a direct comparison of the speech intelligibility of the sentences before and after VSB implantation was possible.

### Self-assessment scales

Quality-of-life assessments were performed using the standardized questionnaires “Speech, Spatial and Qualities of Hearing Scale^®^” (SSQ12) [20] for adults and “Speech, Spatial and Qualities of Hearing Scale^®^ Parents” (SSQP) for children (German version) [21]. The questionnaires document the subjects’/parents’ personal experiences and hearing abilities in various daily life situations with subscales “speech understanding”, “spatial hearing”, and “qualities of hearing”. The assessment was designed to complement the behavioral or experimental measures of hearing ability. In the interest of efficiency, the short form of the SSQ, the SSQ12 [22, 23], designed in 2013 by Noble et al., was used in this study. It consists of 12 questions (instead of 49) from the above-mentioned subscales and provides comparable results to the full version [20].

Both the SSQ12 and the SSQP used a ten-point Likert scale. Adolescents and adults (*n = *33), who had already reached the age of 12 at the time of data collection, were asked to complete the SSQ12. Parents were asked to complete the SSQP for children (*n = *16, < 12 years). In addition, the current daily wearing time of the implant was asked in hours (Assessment by patients or parents).

### Statistical analysis

Descriptive statistics (mean, standard deviation (SD), and range) were calculated to report patient-related characteristics, audiometric outcomes, and the results of the self-assessment questionnaires. Preoperative and postoperative thresholds (BC and AC) and postoperative free-field thresholds (unaided and aided) were compared using two-tailed, paired sample *t* tests. Parametric independent group *t* tests were performed to detect postoperative differences between groups. Correlational analyses (Pearson product moment) were used to examine the relationships between possible influencing factors and speech detection thresholds (SDT).

Statistical significance was set at *p < *0.05. Effect size f was calculated with Cohen. IBM SPSS Statistics 19 for Windows software (Chicago, IL, USA) was used for all analyses.

## Results

### Subjects

Within the included patient group (*n = *51), 42 patients (79.2%; 18 females, 23 males, age 8.9 ± 4.1 years) were under 18 years of age at the time of implantation, 11 were adults (20.8%, 6 females, 4 males, age 32.6 ± 10.5 years). The youngest patient was 5 years old and the oldest patient was 53 years old. As two patients had bilateral atresia of the auditory canal, 53 VSB implantations were performed in 51 patients. The sociodemographic details and specific data of the patient collective are shown in Table 1.

**Table 1:** Demographic and clinical data of the 51 patients

	*n*	%
**Subjects**		
Female	24	47.1
Male	27	52.9
**Age at implantation**		
Children < 18th year of life	42	79.2
Adults > 18th year of life	11	20.8
**Implanted side**		
Left	19	37.3
Right	30	58.8
Both	2	3.9
**FMT site**		
Incus (short process)	12*	22.7
Stapes	36	67.9
Round window	5	9.4
**Grading of hearing loss on implanted ear**		
Mild (21–40 dB HL)	0	0
Moderate (41–60 dB HL)	4	7.5
Severe (61–80 dB HL)	42	79.2
High (> 81 dB HL)	7	13.3
**Diagnosis**		
Congenital atresia	51	100
Unilateral	49	95.9
Bilateral	2	4.1
Caused by		
Singular congenital atresia	5	9.8
Syndromal	5	9.8
With dysplasia of the external ear	41	80.4
**German native language**		
Yes	47	92.2
No	4	7.8
**Prior surgery on implanted ear**		
No	45	84.9
Yes	8	15.1
**Opposite ear**		
Normacusis	44	86.3
Hearing impairment	7	13.7

*One patient was treated with a VSB 502 with modified coupling to the malformed long process of the incus. The clip had detached from the long process, so a conversion to the short incus process using the short process incus coupler was performed.

Eight patients (15.1%) had undergone previous surgery on the implanted ear. Of these, six patients had undergone auricular reconstruction for auricular dysplasia prior to VSB implantation, and 1 patient had undergone meatoplasty on both sides.

#### Follow-up periods

The minimum follow-up (period) of VSB-supplied/received children (*n = *42) was 13 month, the maximum was 157 months (12 years), and average 58.0 (4.8 years). The mean follow-up of VSB-supplied/received adults (*n = *11) was 61.0 months (5.1 years), (range 11 month–127 months (10.6 years)). Since the follow-up visits were not attended by the patients at fixed intervals, the follow-up period was divided into time frames (initial fitting, after 1–3 years, after 3–5 years, and over 5 years). All the patients presented for initial fitting after 2 months, 26 patients (51%) presented 1–3 years after implantation, 23 patients (45%) presented 3–5 years after implantation, and 25 patients (49%) presented for audiometric follow-up more than 5 years after implantation.

At the time of the data collection, surgical complications or device deficiencies were reported in 5 patients (9.4%). FMT dislocation occurred in 2 patients. One patient underwent exploration of the VSB and lysis of adhesions due to symptoms of discomfort. One patient needed repositioning of the FMT coupler due to repeated mastoid irritation. Revision surgery restored regular function of the VSB in all patients.

### Audiological results

#### Comparison of air conduction hearing threshold before and after implantation

From the total of 53 ears tested (*n = *51), the average hearing threshold in the pure-tone audiogram was 70.9 ± 10.2 dB HL distributed over all frequencies (250 Hz–8000 Hz) before surgery. At first fitting, the average hearing threshold without an activated VSB implant was 65.5 ± 10 dB HL. This corresponds to an average improvement of the air conduction threshold hearing gain at initial fitting after implantation of 5.5 ± 7.9 dB HL. The frequency-dependent hearing thresholds remained stable over the long term (Figure A in supplementary). A comparative analysis of the hearing threshold before and after surgery revealed a significant improvement in hearing ability (*t = *5.09, *p < *0.001). The effect size according to Cohen [24] is *r = *0.69, and thus corresponds to a strong effect.

#### Comparison of bone conduction hearing threshold before and after implantation

The values of the preoperative bone conduction threshold (*M* = 14.4, SD = 7.5) are almost identical compared to the postoperative (*M* = 13.3, SD = 5.5), so that there is no significant influence of the implantation on the bone conduction threshold (*t = *1.4 *p* = 0.079). Thus, intraoperatively induced inner ear deterioration can be excluded (Figure B in supplementary).

#### Functional gain

**Fig. 1:**
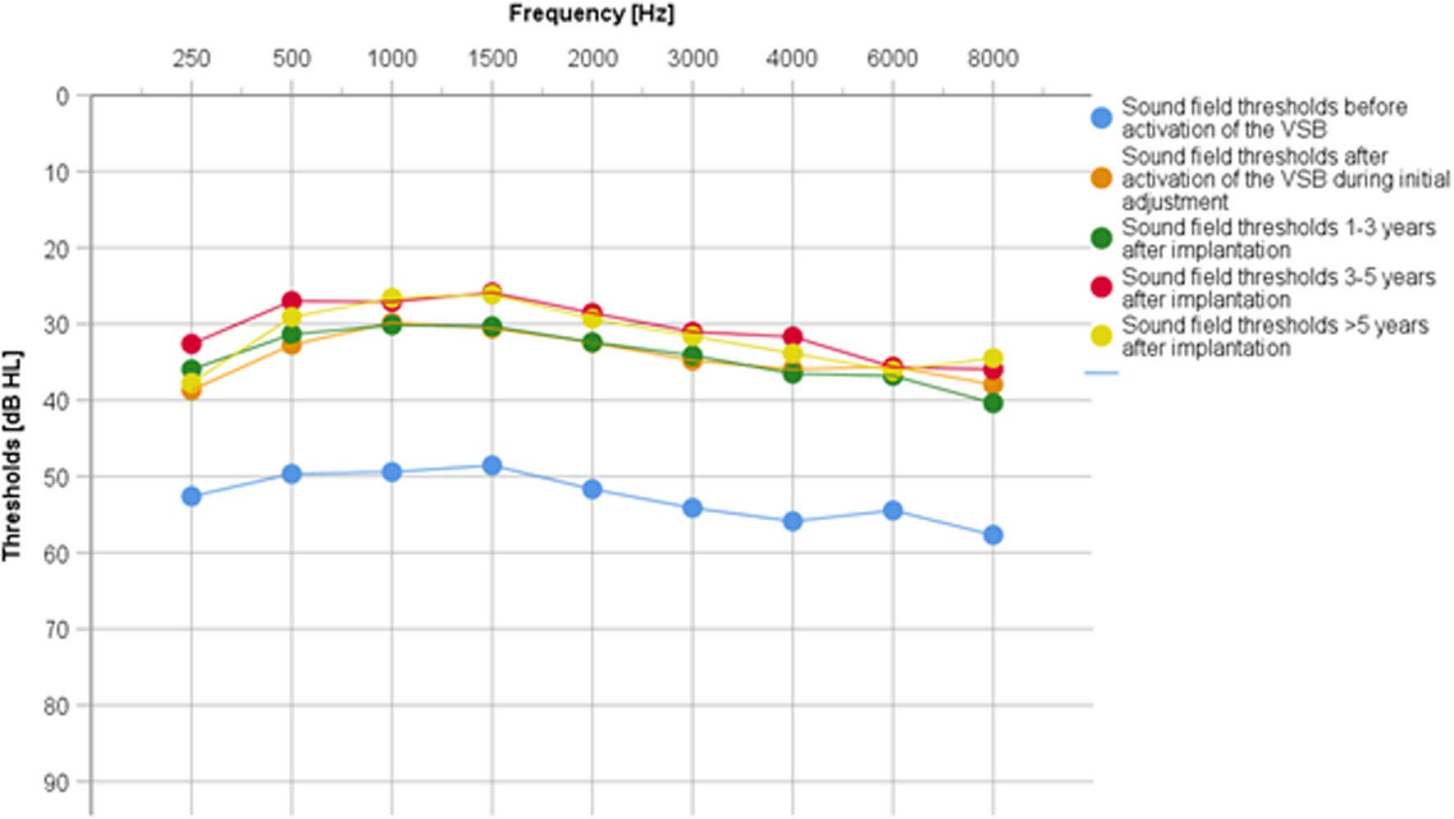
Comparison of hearing thresholds over the long term. Wearing a VSB leads to a significant functional gain with an average of 18.5 dB HL

**Fig. 2:**
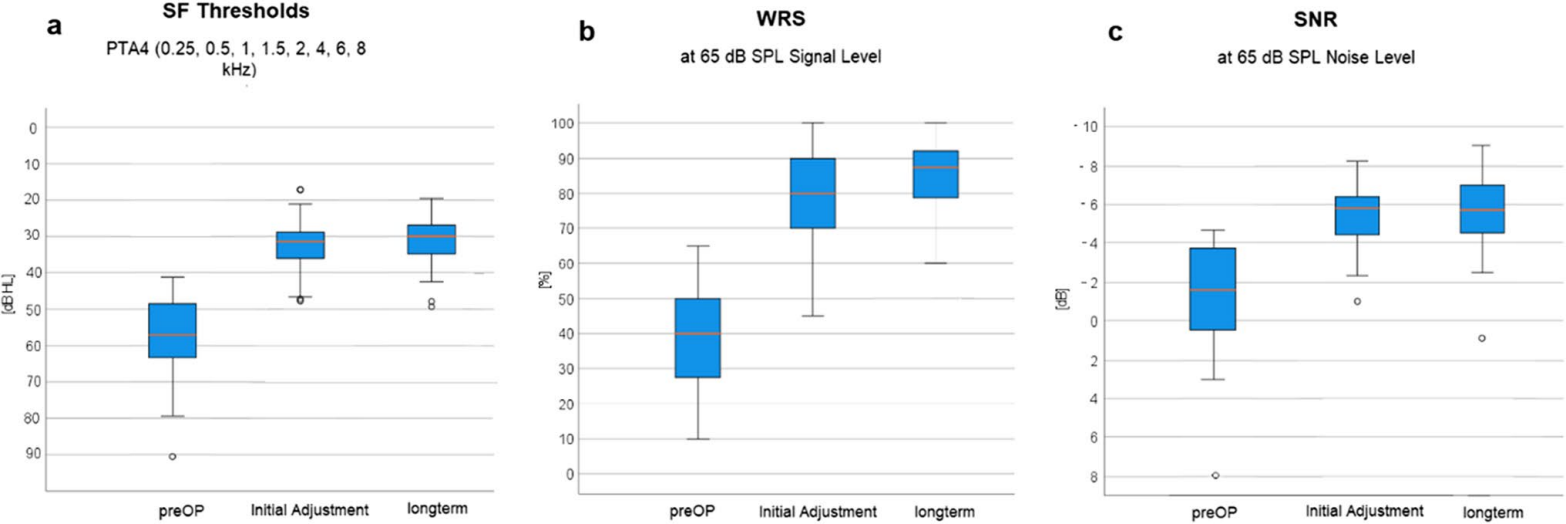
**a** Graphical representation of sound field thresholds [dB HL] using warble tone PTA4 before implantation, at initial fitting and in the long term. (Figure C in supplementary shows the results for patients implanted between 5 and 8 years of age and patients > 8 years of age). **b** Graphical representation of word recognition in a quiet environment (Freiburg monosyllabic speech test in a quiet environment) [dB] before implantation, at initial fitting, and in the long term. With the VSB, there is an improvement of 41.2 ± 13.6%. The scatter of the averaged measured values before implantation with standard deviations of was significantly higher than for the measured values after implantation (values). (Figure D in supplementary shows the results for patients implanted between 5 and 8 years of age and patients > 8 years of age). **c** Graphical representation of speech reception in noisy environments (OLSA/OLKISA) [dB] before implantation, at initial fitting, and in the long term. The deviating value (outlier) at initial fitting was a non-German-speaking patient who may have had problems with word comprehension. (Figure E in supplementary shows the results for patients implanted between 5 and 8 years of age and patients > 8 years of age). The long-term outlier was a patient with cat eye syndrome which can be associated with slightly impaired intelligence, but there is no record of this in the patient's file; so, we consider this result to be most likely the result of a lack of cooperation in the performance of this test

The average hearing threshold in the functional gain curve without VSB distributed over all frequencies (250 Hz–8000 Hz) was 57.5 ± 10.6 dB HL, with VSB at initial fitting 32.6 ± 6.7 dB HL. The long-term hearing threshold was 32.2 ± 7.8 dB HL at 1–3 years after implantation, 30.5 ± 7.9 dB HL at 3–5 years, and 30.8 ± 6.6 dB HL at > 5 years. The average hearing gain was 38.4 ± 9.4 dB HL at first fitting after VSB activation and 39.7 ± 9.5 dB HL over the long term. A stable hearing gain can be observed across all frequencies and over the long term. (Fig. 1 and Fig. 2a).

A single-factor variance analyses with repeated measurements (assumed sphericity: Mauchly-W(2) = 0.716, *p < *0.0.001) with Bonferroni-corrected pairwise comparisons showed that the hearing threshold immediately after implantation (*M* = 32.6, SD = 8.3) was significantly (*p < *0.001) better than before implantation (*M* = 57.5, SD = 10.6).

In contrast, the average hearing thresholds immediately after implantation and in the long-term follow-up were not significantly different from each other (*p* = 1.0), indicating that hearing improvement remains stable over time. The effect size f according to Cohen [24] was 2.8 and corresponds to a strong effect.

#### Word recognition in a quiet environment (Freiburg monosyllabic speech test in a quiet environment)

Word recognition in the Freiburg monosyllable test in a quiet environment averaged 37.5% without VSB at a volume level of 65 dB. After the implantation and fitting of the VSB, word recognition improved to an average of 78.8%. With the VSB there is an improvement of 41.2 ± 13.6%. Over the long term, word recognition remained stable at a similarly high level (*x̅* = 84.2%) (Fig. 2b).

The paired *t* test showed significant improvements of the measured values before and after implantation with *t = *21.68, *p < *0.001. However, there was no significant difference between the results at initial fitting and in the long term (*t = *0.74, *p* = 0.23). The Bonferroni-corrected pairwise comparisons of the analysis of variance confirmed the significance of the measured values before and after implantation (*p < *0.001). This shows a long-term, stable hearing improvement.

#### Speech reception in noisy environments (OLSA/OLKISA)

Speech reception in OLSA/OLKISA before implantation showed wide scatter between the individual patients. The maximum value was − 5.85 dB S/N, while the minimum value was 9 dB S/N. After implantation, there was an average improvement in speech reception from − 0.9 dB S/N to − 5.2 dB S/N. A sustained effect was also demonstrated in the long-term (course) with a mean value of − 5.4 dB S/N (Fig. 2c).

The paired *t* test comparisons showed a significant improvement in speech comprehension in noisy environments after implantation (*t = *10.887, *p < *0.001). In contrast, speech comprehension immediately after implantation and in the long term did not differ significantly (*t = *0.439, *p* = 0.332).

#### Self-assessment scales

**Fig. 3:**
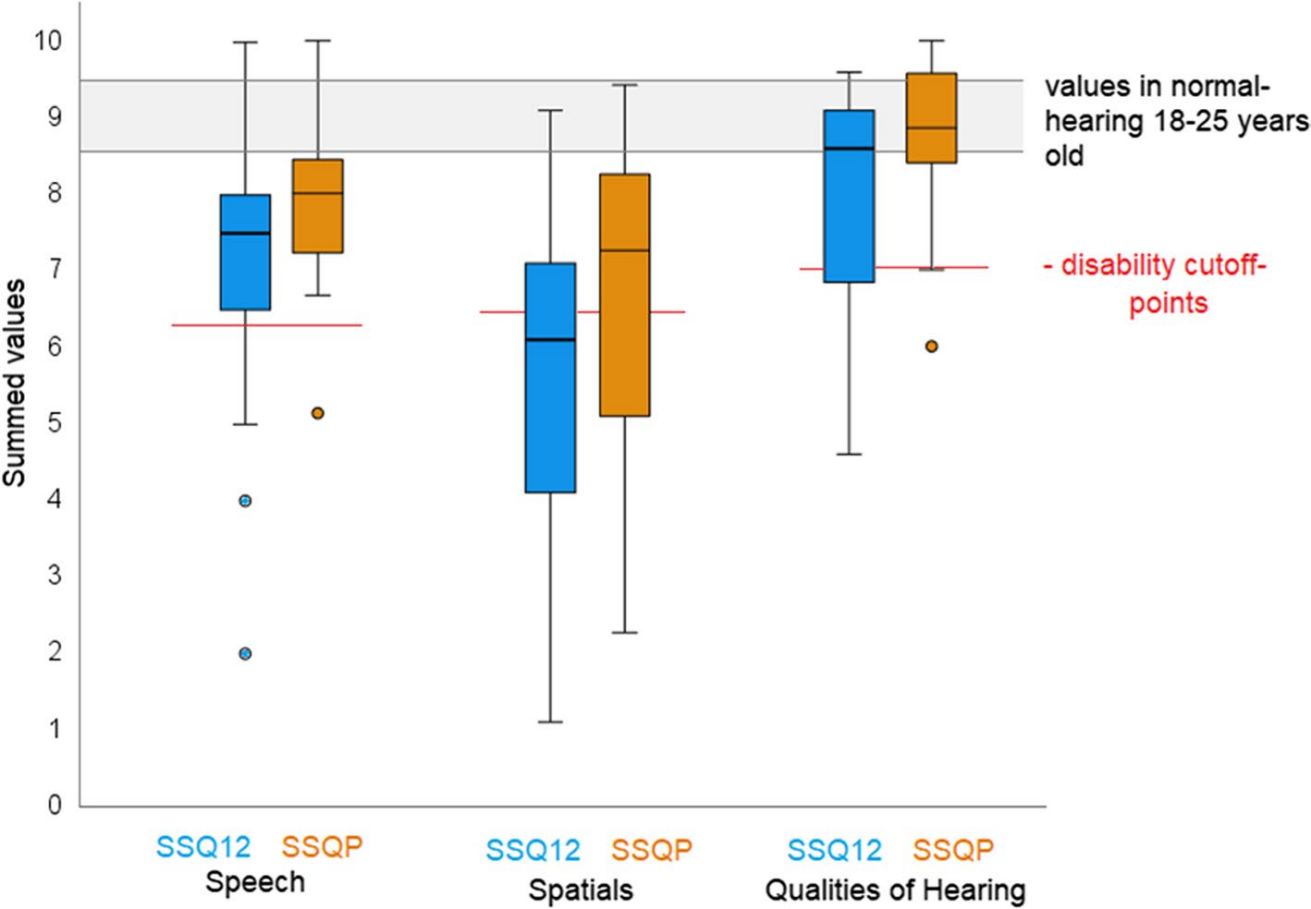
The twelve questions of the SSQ12 were examined with respect to the subscales “speech understanding” (questions 1–5), “spatial hearing” (questions 6–8), “qualities of hearing” (questions 9–12). The arithmetic mea*n = x̅*, marked in orange can also be taken from the boxplots: The 23 questions of the SSQP were examined with respect to the dimensions “speech understanding” (questions 1–9), “spatial hearing” (questions 1–6), “hearing quality” (questions 1–8). The arithmetic mea*n = x̅*, marked in black can also be taken from the boxplots. Scores of young people with normal hearing (age 18–25 years) described by Demeester et al. (gray background), they reached a mean score between 8.5 and 9.3. Cut-off values for disability (red lines) [35]

The response rate to the survey by questionnaire was 100 percent. The 12 questions of SSQ12—answered by 35 patients—can be assigned to three subscales [25]. The subscale “speech understanding” (questions 1–5, for example: *“You are talking with one other person and there is a TV on in the same room. Without turning the TV down, can you follow what the person you’re talking to says?”)* was rated on average with a value of 7.3 (SD 1,6). The subscale “spatial perception” (questions 6–8, for example: *“Can you tell how far away a bus or a truck is, from the sound?”)* was rated on average with a value of 5.7 (SD 1,9) and the subscale “clarity, separation, and identification” (questions 9–12, for example: *“When you listen to music, can you make out which instruments are playing?”)* with a value of 7.9 (SD 1,3). The results of the cumulative frequencies are presented in Fig. 3.

The 23 questions of SSQP—answered by 16 patients’ parents—can be also assigned to three subscales [21]. The subscale "speech understanding" (questions 1–8, for example: *“You are talking with your child and there is a TV on in the same room. Without turning the TV down, can your child follow what you’re saying?”)* was rated on average with a value of 7.9 (SD 1.2). The subscale “spatial hearing” (questions 1–5, for example: *“You and your child are outside. You call out their name. Can your child tell immediately where you are without having to look?”)* was rated on average with a value of 6.8 (SD 2.1) and the subscale “hearing quality” (questions 1–8, for example: *“You are in a room with your child and music is playing. Will your child be aware of your voice if you start speaking?”) with a value of 8.2 (SD 1.0).* The results of the cumulative frequencies are presented in Fig. 3.

#### Wearing time

Some of the study participants reported very large differences in the wearing time of the VSB per day. 22 patients (41.5%) reported a daily wearing time of 5–10 h, and 24 patients (45.3%) a daily wearing time of 10–15 h. 3 patients (5.7%) reported an average wearing time of < 5 h/day, and 3 patients (5.7%) an average wearing time of > 15 h/day. Because the latter two numbers of cases are too small for a statistically significant statement, they were not considered in the following analysis of variance.

#### Factors influencing the audiological outcome and the quality of life after VSB implantation

The wearing period did not significantly affect the long-term audiological outcome in the functional gain curve (*r = *0.049, *p* = 0.754, *n = *44), the Freiburg monosyllabic speech test a in quiet environment (*r = *0.029, *p* = 0.845, *n = *49), or the Oldenburg sentence test (*r = *0.050, *p* = 0.729, *n = *50).

Similarly, there was no significant correlation between the age of a patient at implantation and postoperative audiological outcome (functional gain: *r = * − 0.058, *p* = 0.678, *n = *53, Freiburg monosyllabic speech test in a quiet environment: *r = * − 0.171, *p* = 0.252, *n = *47; OLSA test: *r = * − 0.110, *p* = 0.440, *n = *51). Consequently, patients of several ages benefit from VSB implantation.

The subscales “speech understanding” and “hearing quality” of the questionnaires and the averaged postoperative speech comprehension in the Freiburg speech test and OLSA did not significantly correlate. Since no audiological test for directional hearing was performed in the study, no statement can be made about a correlation with the subgroup “spatial hearing”.

Hearing gain in the free-field audiometry, in the Freiburg speech test and in the OLSA test correlated significantly with audiological performance before implantation (free field: *r = *0.750, *p < *0.001, *n = *53), (Freiburg speech test: *r = *0.762, *p < *0.001, *n = *53), (OLSA test: *r = *0.761, *p < *0.001, *n = *53). Consequently, patients with a poorer starting level benefited most from implantation. This was a strong effect according to Cohen (*r* > 0.5) [24].

## Discussion

This study analyzed and compared the audiological and the self-assessed hearing performance of 51 patients with VSBs over a period of 12 years. The results indicated a stable functional gain provided by the VSB in the frequency range 125–8000 Hz. All patients included in this study benefited significantly from the VSB. This benefit was demonstrated both in the hearing performance, investigated by pure-tone and speech recognition audiometry, and in patients’ self-assessments, evaluated by standardized questionnaires. To date, it presents one of the largest studies of children, adolescents, and adults with aural atresia who received a VSB and audiometric long-term follow-ups for years.

### Study design

Many previous studies investigating audiological outcome in patients with atresia of the auditory canal only report on a follow-up period after initial fitting, which means approximately six weeks after implantation. [3, 9, 26–28] or up to 3 or 6 months after implantation [29–31]. Hempel et al. [32] and Mandala [33] reported long-term results up to 36 months and 41.7 ± 18.6 months on average, respectively. The present study has one of the longest follow-up to date in these patients, with a mean follow-up of 58.6 months or 4.8 years.

### Hearing tests

The results show no significant BC threshold shift after surgery. Thus, there was no intraoperative inner ear deterioration, e.g., due to possible iatrogenic traumatization of the conductive apparatus or due to excessive noise exposure during milling in the mastoid. All patients achieved socially useful hearing in terms of free-field audiometric thresholds and speech reception at a conversational level. [9].

The FMT coupling site did not influence the audiological outcome in this study or in comparable studies [34–36, 39].

Ear surgery preceding VSB implantation did not significantly influence audiological outcome after VSB implantation, as these were operations that did not involve structures relevant to VSB coupling and subsequent transmission of signals to the inner ear, such as auricular reconstructions. After VSB implantation, there was an average improvement in the air conduction threshold of 5.5 ± 7.9 dB which was stable over the long term. VSB implantation had no effect on air conduction hearing threshold in Claros et al., Mandala et al. nor Zernotti et al. [9, 28, 33]. This minor hearing threshold improvement is most likely due to surgery. Drilling away bone material, loosening adhesions, and coupling the FMT result in improved ossicular vibratory capacity and, thus, improved conduction hearing threshold in air conduction measurements.

#### Functional gain curve

The functional gain curve with VSB showed a significant functional hearing gain of 38.4 ± 9.4 dB HL at first fitting, and 39.7 ± 9.5 dB HL over the long-term dB on average, so the hearing threshold remains approximately constant. In the frequency-specific analysis of the functional hearing gain, it was noticeable that there was an almost identical gain in function at all frequencies, and that this gain was stable over the long term. These results confirm the findings of Hempel et al. [32], who found an average functional hearing gain of 29.49 ± 13.18 dB HL in 31 patients, the findings of Mandala et al. (2011), who found an additional gain of 11–34 dB HL in 14 children [33], and the results of Attaway et al. [83], which showed an average gain in function of 22.3 dB HL. Thus, the results reported here can be categorized within the range of 12.9 dB–47.2 dB described in a meta-analysis with a total of 796 patients in the middle range [34], and are also within the reported range of 18–40 dB HL (*x̅* = 28.7 dB HL) [97].

Comparable data were also presented by Roman et al. [27] in 10 children with atresia of the auditory canal, who showed a hearing gain of 38 dB HL, and Célérier et al. [17], who showed a hearing gain of 39 dB in 3 children with microtia.

Probably, the highest hearing gain with 45.5 and 48 dB HL was measured by Frenzel et al. [26, 30]. However, these studies only included 7 and 4 subjects with unilateral congenital atresia of the auditory canal [75, 86].

Word recognition (Freiburg monosyllabic speech test in a quiet environment).

The hearing gain shown in the pure-tone audiograms was also demonstrated in the speech comprehension tests. In this study, the average word recognition in a quiet environment with VSB at 65 dB HL sound pressure was 84.8%. This corresponds to a 41.2% improvement in speech comprehension with the VSB, and is as good a result as the comparable result from the study by Frenzel et al. [30] which described a gain of 38%.

Frenzel et al. published a study on seven atresia patients implanted with the AMEI, with a WRS of 99% and Hempel et al. with a WRS of 95.38% in children and 84.71% in adolescents.

A meta-analysis with 195 patients showed a wide range from 55% up to 95% word recognition [37]. This can be explained by the different initial levels before implantation—from 0 up to 72% in this study.

Word recognition before implantation correlated significantly positively with outcome after implantation, i.e., patients with a worse starting level benefit more from VSB implantation.

#### OLSA/OLKISA

A significant and stable improvement of speech reception in noisy environments (OLSA/OLKISA) with the VSB implant was found. Speech reception in noisy environments before implantation correlates significantly with outcome after implantation, i.e., patients with a poorer starting level benefit more from VSB implantation. An improvement of the hearing situation in everyday life can be derived from this. The average gains in the study by Frenzel et al. [29] in speech reception for 5–9-year-olds with 5.2 dB SNR, and 6.4 dB SNR for 10–17-year-olds, correspond with the average gain of 6.4 dB S/N found here, and with 6.9 dB SNR found by Hempel et al. [32]. In total, only three studies to date have been conducted to measure speech comprehension in VSB-implanted patients using the German Oldenburg Sentence Test (OLSA/OLKISA).

### Questionnaires

In addition to the improvement in the audiometric tests, there was also an improvement in hearing quality in everyday situations in the subjective assessment of quality of life using SSQ12 and SSQP.

The VSB-supplied adolescents and adults achieved a mean score of 6.8, 5.7, and 7.7, the children scored 7.9, 6.7, and 8.1 in the three subscales “Speech”, “Spatial Hearing”, and “Hearing Quality”. Comparing these results with the scores of young people with normal hearing (age 18–25 years) described by Demeester et al., it can be seen that they reach a mean score between 8.5 and 9.3 [38]. Significant impairment was seen in young people with normal hearing (18–25 years) with scores below 6.1, 6.2, and 6.8 in the subscales “Speech”, “Spatial Hearing”, and “Hearing Quality” [38]. Thus, only spatial hearing shows a significant impairment for VSB patients compared to patients with normal hearing (Fig. 3).

### Wearing time/period per day

95% of the patients wear the audio processor for more than 5 h per day. These results confirm the safety and the effectiveness of the VSB in a long-term follow-up. The wearing time, implantation age, or previous surgery on the implanted ear had no significant influence on the functional gain curve.

In addition to the patients (*n = *51) included in the study, there were 3 non-users who did not want to participate in the study. Accordingly, no data from these 3 patients could be included in the study. Compared with the patient population in the study by Cladre et al. [39], this represents a much lower proportion.

## Limitations

Due to the retrospective data collection for the study, follow-up was divided into different time intervals as patients presented for check-ups at different intervals. Not all patients could be followed up over the same uniform time period due to the long interval between implantations.

Generally, the international comparison of the study design and the results is limited. None of the publications published reported a follow-up period for the children or adults as long as the study presented here. Furthermore, there are variations in the patient group, in audiometric measurements, and in the questionnaires used for measuring patient satisfaction or quality of life. In particular, the comparison of speech recognition is limited, because there is no international standardization for speech audiometry. However, the audiometric and patient satisfaction results can be globally compared with other retrospective studies. The SSQ12 is used internationally, so the results are clinically comparable worldwide and immediately provide an objective statement.

Possible side effects such as aural fullness or complications such as acoustic trauma due to over-stimulation, fibrosis surrounding the ossicles, erosion of the long process, device failure with reimplantation or explantation were not observed or measured/tested. Consequently, no statement can be made about these situations in the long term.

However, dislocation of the FMT occurred in two patients with no apparent cause. During surgical exploration, damage to the stapes superstructures or fracture of the stapes legs was excluded as the cause of the dislocation. One patient was treated with a VSB 502 with modified coupling to the malformed long process of the incus. The clip had detached from the long process, so a conversion to the short incus process using the short process incus coupler was performed.

The second patient initially had a clip coupler on the malformed stapes. After spontaneous dislocation, coupling was performed at the round window.

Another patient underwent surgical exploration and lysis of adhesions for recurrent middle ear infections after VSB implantation. Impairment of middle ear ventilation due to the short incus coupler complex might have been responsible as the cause of the recurrent inflammations. The new coupling on the stapes led to permanent freedom from complaints.

In another patient who was treated with a short process incus coupler, an acute mastoiditis occurred 6 weeks postoperatively. A revision was made with taking the coupler from the incus and putting the FMT on the stapes using clip coupler with the assumption that the ventilation in the area of the upper ventilation line was restricted by the SPI Coupler with FMT. This complication could be completely eliminated, so the hearing results after the revision corresponded to the measured values at the initial adjustment.

Additional long-term studies are necessary to confirm the rate of complications. Mosnier et al. [10] observed no adverse effects for more than 5 years. These results confirm the safety and the effectiveness of the VSB with a long-term follow-up.

One special case was an adult patient with ear canal reconstruction and autologous incus interposition for ossicle reconstruction in childhood. Initial coupling of the FMT was to the round window. After deterioration of the inner ear function over a period of about five years, a conversion to the stapes was performed with removal of the autologous incus interponate, which was fused to the stapes. During the initial implantation of the VSB, the risk of stapes dislocation was considered too high with the above-mentioned ossification. Prior to revision surgery of the VSB, the patient was informed that in case of stapes luxation, a cochlear implant would have to be used.

In general, the decision to FMT coupling was made as follows: Whether the incudostapedial joint existed with a good mobility, coupling was performed onto the short process of the incus; if the hammer and incus were fixed, coupling was performed onto the stapes. The operative procedure is described in Braun et al. [40]. Patients with a Jahrsdoerfer score of 5 or better were treated with a VSB.

A subanalysis, whether the type of coupling influences the audiological result, was not made as there was no significant differences relating to the functional gain or speech comprehension in prior studies Claros et al. [9, 39].

In everyday situations, people usually listen binaurally. The extent to which hearing impairment of the opposite ear and a hearing aid fitting influences the overall hearing impression was not investigated in this study.

Directional hearing was only assessed by the SSQ12/ SSQP, other aspects in determining quality of life, such as psychological and social factors, were not determined.

## Conclusion

This study analyzed and compared the audiological and the self-assessed hearing performance of 51 patients with VSBs over a period of 12 years. The results indicated a stable functional gain provided by the VSB and a good tolerance of the implant. The benefit was demonstrated both in the hearing performance, investigated by pure-tone and speech recognition audiometry, and in patients’ self-assessments, evaluated by standardized questionnaires. To date, it presents one of the largest studies of children, adolescents, and adults with aural atresia who received a VSB and audiometric long-term follow-ups for years.

### Supplementary Information

Below is the link to the electronic supplementary material.

Supplementary Figure A The frequency-dependent hearing thresholds remained stable over the long term file1 (PNG 48 KB)

Supplementary Figure B The frequency-dependent hearing thresholds remained stable over the long term file2 (PNG 33 KB)

Supplementary Figure C SF thresholds (PTA4 (0.25, 0.5 1, 1.5, 2, 4, 6, 8 kHz) for patients implanted between 5 and 8 years of age and patients > 8 years of age. file3 (PNG 26 KB)

Supplementary Figure D WRS at 65 dB SPL signal level for patients implanted between 5 and 8 years of age and patients > 8 years of age file4 (PNG 17 KB)

Supplementary Figure E SNR at 65 dB SPL signal level for patients implanted between 5 and 8 years of age and patients > 8 years of age. file5 (PNG 20 KB)

## Data Availability

Data available on request from the authors.

## Data materials and/or code availability

Data are available from the authors.
