# Supplementary figures and images for: Long-term results and quality of life after vibrant soundbridge implantation (VSBs) in children and adults with aural atresia
Source: Eur Arch Otorhinolaryngol. 2023 Aug 21;281(1):129–39. doi: 10.1007/s00405-023-08100-y (PMC10764446; doi:10.1007/s00405-023-08100-y)

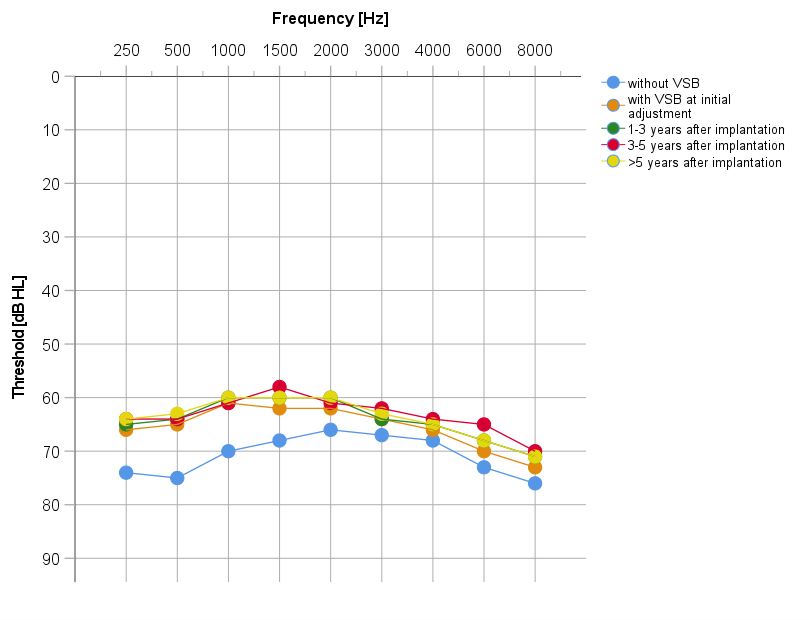

Supplement: Supplementary file 1 — Supplementary Figure A The frequency-dependent hearing thresholds remained stable over the long term file1 (PNG 48 KB) [file 405_2023_8100_MOESM1_ESM.png]

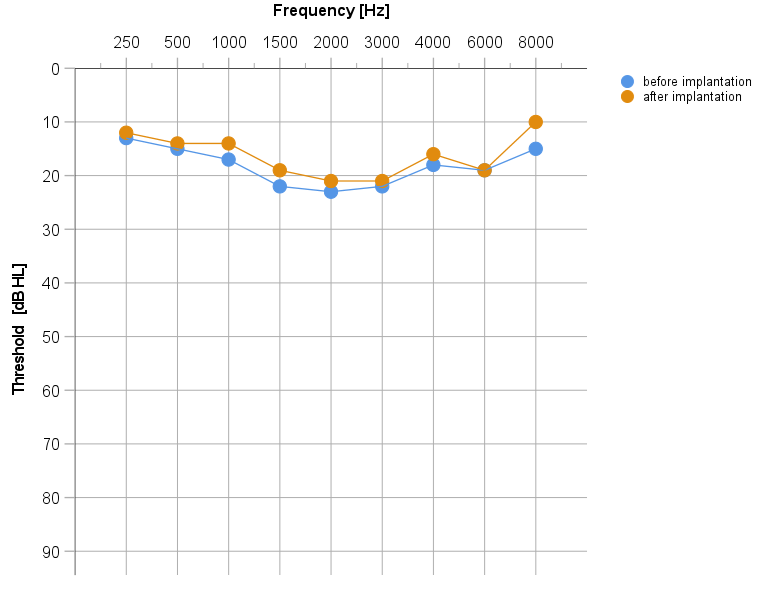

Supplement: Supplementary file 2 — Supplementary Figure B The frequency-dependent hearing thresholds remained stable over the long term file2 (PNG 33 KB) [file 405_2023_8100_MOESM2_ESM.png]

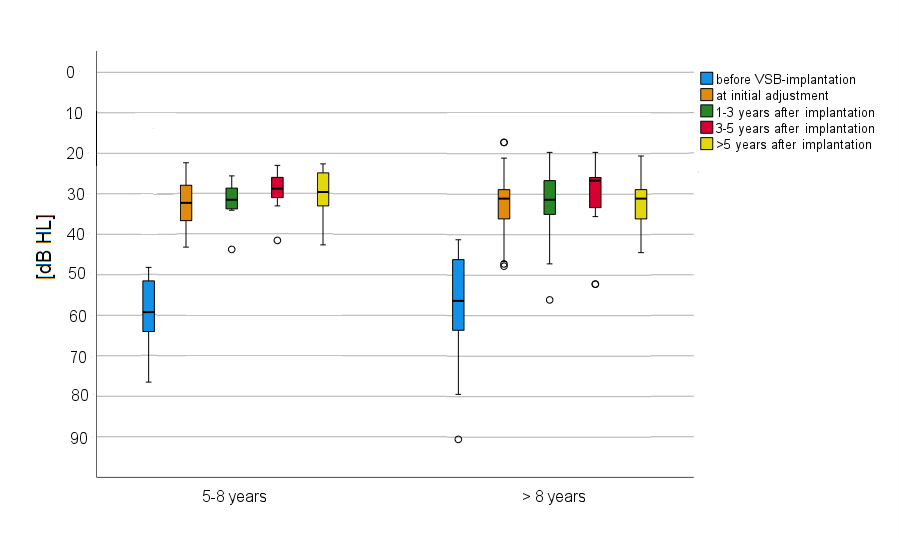

Supplement: Supplementary file 3 — Supplementary Figure C SF thresholds (PTA4 (0.25, 0.5 1, 1.5, 2, 4, 6, 8 kHz) for patients implanted between 5 and 8 years of age and patients > 8 years of age. file3 (PNG 26 KB) [file 405_2023_8100_MOESM3_ESM.png]

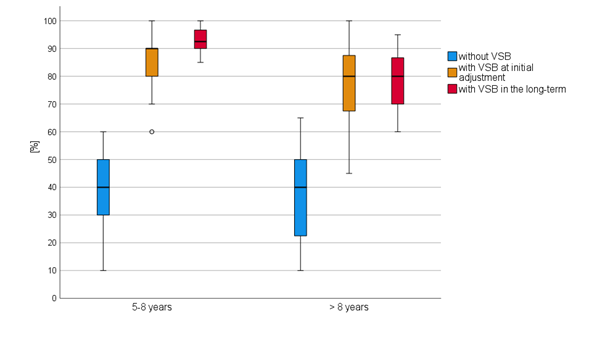

Supplement: Supplementary file 4 — Supplementary Figure D WRS at 65 dB SPL signal level for patients implanted between 5 and 8 years of age and patients > 8 years of age file4 (PNG 17 KB) [file 405_2023_8100_MOESM4_ESM.png]

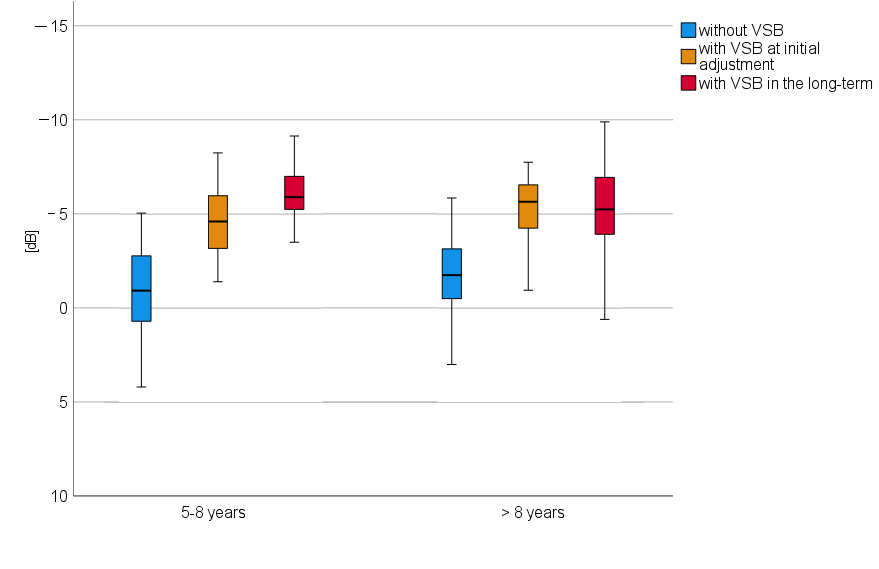

Supplement: Supplementary file 5 — Supplementary Figure E SNR at 65 dB SPL signal level for patients implanted between 5 and 8 years of age and patients > 8 years of age. file5 (PNG 20 KB) [file 405_2023_8100_MOESM5_ESM.png]
